# Real-Life State of Anti-Hepatitis B Virus Drug Choice in Child-Bearing Age Male Patients and Effect on Fertility and Fetal Safety

**DOI:** 10.1155/2019/9703907

**Published:** 2019-04-01

**Authors:** Zhao X. Hu, Yi N. Ye, Wei G. Wu, Xu J. Liang, Qi W. Wu, Ao Zhang, Xing R. Zheng, Zhi L. Gao, Liang Peng, Chan Xie

**Affiliations:** ^1^Department of Infectious Diseases, The Third Affiliated Hospital of Sun Yat-Sen University, Guangzhou, Guangdong Province, China; ^2^Department of Infectious Diseases, The First People's Hospital of Foshan, Foshan, Guangdong Province, China; ^3^Key Laboratory of Infection and Immunity, Institute of Hepatology, Shenzhen Third People's Hospital, Shenzhen, Guangdong Province, China; ^4^Department of Infectious Disease, The First Affiliated Hospital, Jinan University, Guangzhou, Guangdong Province, China; ^5^Department of Infectious Disease, Zengcheng People's Hospital, Guangzhou, Guangdong Province, China; ^6^Department of Nephrology, Guangdong Second Traditional Chinese Medicine Hospital, Guangzhou, Guangdong Province, China; ^7^Guangdong Provincial Key Laboratory of Liver Disease, The Third Affiliated Hospital of Sun Yat-Sen University, Guangzhou, Guangdong Province, China

## Abstract

Research on effects of anti-hepatitis B virus (HBV) nucleoside analogs on male fertility and birth defects is limited and safety of nucleoside analogs in pregnancy is still a concern. Chronic hepatitis B (CHB) patients in Guangdong province were surveyed using a structured questionnaire. We collected data including medication type, fertility, and birth defects. Moreover, a survey of the knowledge of antiviral nucleoside analogs safety in fertility of male patients was conducted among physicians nationwide. Semen samples of 30 patients were collected. We screened 1050 HBV-positive male patients. Reasons for not receiving antivirals in 150 patients were “did not meet criteria for antiviral therapy,” fertility, and financial. Furthermore, 900 participants received antivirals (85.71%, 900/1050), including 792 patients with children and 15.15% (120/792) took anti-HBV treatment when preparing for pregnancy. Based on whether they received antiviral therapy during conception or not, we divided patients into two groups. In the child-bearing age group, 88.33% (106/120) of patients received telbivudine (LDT), whereas the other group mainly received entecavir (ETV) (87.20%, 586/672). No significant difference occurred in birth defect incidence rates between both groups. Furthermore, 558 physicians completed questionnaires. Reasons that influenced drug selection were “patient's condition,” “fertility demand,” “financial condition,” and “compliance.” Telbivudine was the first-choice drug (32.80%, 183/558) while tenofovir (TDF) was the second (2.69%, 15/558). Additionally, 61.47% of physicians considered telbivudine or tenofovir as the first choice for male patients who met antiviral criteria, whereas 19% suggested delayed therapy and follow-up until childbirth. No significant changes occurred in semen volume, concentration, mobility, and percentage before and after administration of anti-HBV nucleoside analogs, which did not affect male fertility and birth defect incidence while the desire for pregnancy influenced drug selection and timing of administration. Further research on the effects of analogs on male fertility and fetal safety is required.

## 1. Introduction

Despite substantial progress in global hepatitis B virus (HBV) immunization programs over the past two decades [1], chronic HBV (CHB) infection and its complications, cirrhosis and hepatocellular carcinoma (HCC), remain major public health problems, especially in male patients [2]. Approximately 400 million people worldwide are chronically infected with HBV, with an estimated 4.5 million new infections yearly [3]. Nucleoside analogs are the most widely prescribed antiviral agents, and are safe and effective for chronic HBV [4]. Numerous clinical studies on the efficacy and safety of nucleoside analogs in HBV patients, including exceptional patient groups such as pregnant women and children, have been performed [5]. However, the effect and safety of antiviral nucleoside analogs in child-bearing age male patients has not been well studied.

Antiviral nucleoside analogs inhibit HBV replication by targeting viral reverse transcriptase or polymerase [6]. They simulate natural nucleoside structure and competitively act on central enzyme activity in the polymerase synthesis process [7]. However, it is very difficult to completely eliminate HBV and requires long-term treatment. In China, 7% of the population is HBV S antigen (HBsAg) positive and approximately 20 million are child-bearing age male patients or plan to have children during their spouse's HBV therapy. Therefore, pregnancy safety is a major concern for both doctors and patients during nucleoside/nucleotide analogs treatment because of the latent risks for pregnancy and fetal growth.

In this study, we conducted a questionnaire survey of child-bearing age male HBV patients and liver disease specialists, to understand the toxicity of antiviral agents on male reproduction and the current knowledge of physicians on antiviral nucleoside analogs effect on male fertility, birth defects, and drug selection. The aim was to better manage child-bearing age male HBV patients, optimal treatment selection, and timing of administration.

## 2. Participants and Methods

### 2.1. Case Selection and Study Design

This study was conducted with HBV-positive male patients in the Infectious Disease Department or Liver Disease Department of Guangdong Province's hospitals, including the Third Affiliated Hospital of Sun Yat-Sen University, the First People's Hospital of Foshan, the Third People's Hospital of Shenzhen, the First Affiliated Hospital of Jinan University, and the First People's Hospital of Zengcheng. The inclusion criteria were age between 18 and 50 years and continuous evidence of positive HBV marker or positive HBV DNA for > 6 months. The exclusion criteria were patients with liver cirrhosis, HCC, or concurrent diagnosis of other severe vital organ diseases and those whose wives had HBV infection. Antiviral therapy during the conception period was defined as a man taking antiviral therapy for ≥ 6 months while preparing for pregnancy. Fertility ability examinations were performed before and 6 months after the administration of anti-HBV nucleoside analogs in 30 CHB patients without varicocele, renal disease, hepatic disease, hematological disease, hormonal disorders, genetic disorders, erectile dysfunction, infection, and testicular trauma. Prior to the use of the patients' clinical information for research purposes, their consent was obtained as well as approval from the Institutional Research Ethics Committee of the Third Affiliated Hospital of Sun Yat-Sen University. We collected information such as demographic data, past medical history, family history, personal history, duration of antiviral treatment, antiviral medicine type, fertility, and health condition of offspring of HBV patients receiving antiviral nucleoside analogs (lamivudine (LAM), adefovir dipivoxil (ADV), LDT, ETV, and TDF). We also collected data of untreated patients to record the reasons why they did not receive antiviral nucleoside analogs.

In November 2015, we used the WeChat platform to conduct a questionnaire survey of liver disease specialists nationwide. The questionnaire included plan and treatment approach for child-bearing age male patients, including considerations in deciding a treatment plan for patients who met the criteria for antiviral therapy, the priority and drug of choice, knowledge of effects of antiviral nucleoside analogs on fertility of the patients, and anti-HBV treatment regimen recommendation for patients who met antiviral therapy criteria and wished to conceive in the near future.

### 2.2. Semen Sample Collection and Tests

Semen samples were collected at the beginning of the study for 6 months of anti-HBV drug administration. Sperm samples were collected by masturbation after 3-5 days of sexual abstinence and stored in a plastic container. Then it was incubated at 37°C for 30 minutes and was analyzed after 1 hour. Ethical approval was not required as all the procedures were in vitro and no intervention was conducted on the patients. Written informed consent was collected from all subjects before beginning the study and those not consenting to participate in the study were excluded. Immediately after sample collection, initial semen analysis was performed on a part of each and samples that were normal in terms of the World Health Organization (WHO) 2010 criteria (sperm count ≥15 million/mL, total motility≥40%, progressive forward motility≥32%, normal morphology≥40%, seminal volume 1.5 mL, pH≥7.2, normal appearance and viscosity, and maximum liquefaction time of 1 h) were selected and statistically analyzed. Sperm motility was determined using a computer-assisted sperm analysis (CASA) system. The sperm morphology was determined using the Diff-Quick staining technique and CASA system.

### 2.3. Statistical Analysis

The data analysis was performed using the statistical package for the social sciences (SPSS) version 12 (SPSS Corporation, Chicago, IL, USA). Based on whether the patients conceived children during antiviral nucleoside analogs therapy, they were divided into the conceived and the nonconceived groups. Measurement data are expressed as the mean±standard deviation (SD). Comparison between the groups was conducted using the* t*-test, and enumeration data were compared using the Chi-squared (*χ*^2^) test. Differences were considered statistically significant at* P*<0.05. For the multiple and sorting answers, the mean options comprehensive score (MOCS) was used (MOCS= [Σ frequency × weight value]/number of answers].

## 3. Results

### 3.1. Reasons for Not Taking Antiviral Therapy among Male HBV-Positive Participants

**Figure 1:**
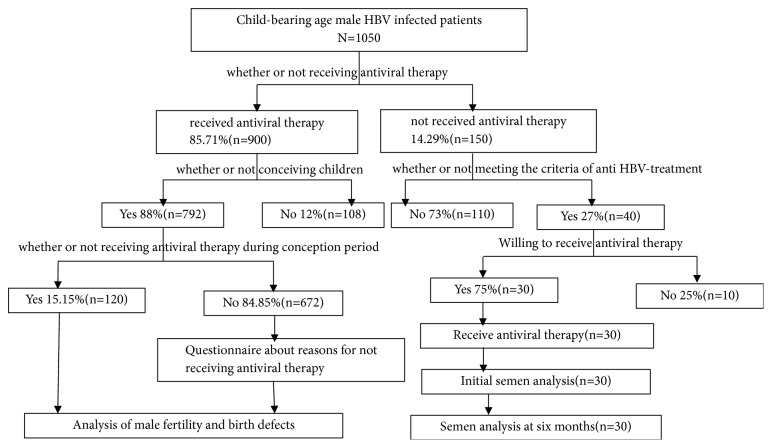
Study design and patient screening flow chart. We enrolled 1050 patients with HBV infection and 30 patients were assigned to anti-HBV therapy and semen analysis. HBV: hepatitis B virus.

**Figure 2:**
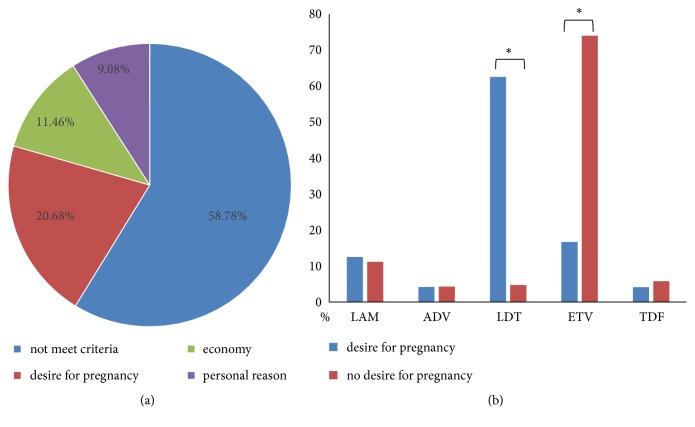
State of antiviral therapy in male chronic hepatitis B virus (HBV, CHB) patients. (a) Main reason why male HBV-positive participants did not receive antiviral treatment. (b) Analysis of nucleoside analog drug distribution in patients with or without desire for pregnancy.

A total of 1050 HBV-infected patients were screened (Figure 1). The median age of patients was 38 years. Based on whether the patient received antiviral therapy or not, the patients were divided into the antiviral and the nonantiviral groups. Among them, 150 HBV-positive patients never received antiviral therapy, while 900 received antiviral nucleoside analogs (85.71%). A total of 120 patients conceived children during treatment and 672 male HBV-positive participants did not take antiviral therapy while preparing for pregnancy. The main reason was “did not meet criteria for antiviral treatment” (58.78%), while 20.68% of the patients did not receive antiviral therapy because they desire a pregnancy (Figure 2(a)).

### 3.2. Nucleoside Analog Drug of Choice for Patients with Desire for Pregnancy

According to the patient's desire for pregnancy during antiviral therapy or therapy preparation, we divided the patients into two groups, those who did and did not desire pregnancy in 3 years. The results showed that the second group mostly received ETV (73.95%), while the first group mostly received LDT (62.50%, Figure 2(b)).

### 3.3. Effect of Antiviral Therapy on Male Fertility and Birth Defects

**Figure 3:**
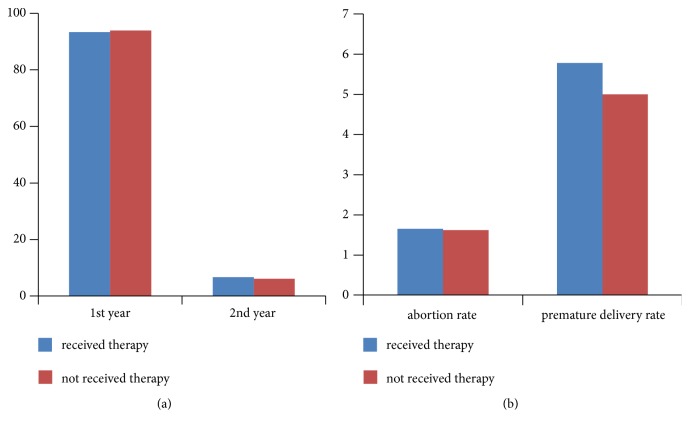
Effect of antiviral therapy on male fertility and birth defects. (a) Impregnation ability and (b) abortion and premature delivery rates in the two groups of patients.

In the research group, 120 patients received antiviral therapy during pregnancy, with 121 newborns. In the research group, 93.33% patients did not use contraception, had a normal sex life, and conceived within 1 year, while 6.67% (8/120) conceived within 2 years. In the control group, 672 patients had 680 newborns. Among these patients, 93.90% (631/672) did not use contraception, had a normal sex life, and conceived within 1 year, while 6.10% (41/672) conceived within 2 years (Figure 3(a)). The abortion and premature delivery rates in the two groups showed no significant difference (Figure 3(b)). There was one case of *β* thalassemia minor in the research group newborns. There was one case of supernumerary finger, three cases of *β* thalassemia intermedia, one enorchismus, and one atrial septal defect in the control group newborns. There was no significant difference in birth defect incidence rates between the two groups (*P*>0.05). None of the offspring in both groups had HBV infection.

**Figure 4:**
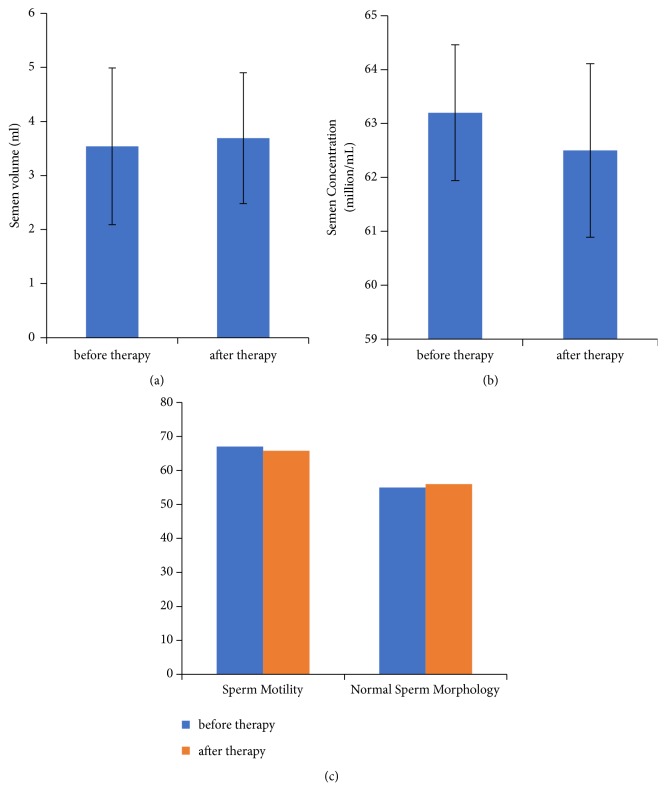
Effect of antiviral therapy on semen and sperm. (a) Semen volume changes in CHB patients before and after taking anti-HBV therapy. (b) Semen concentration and sperm changes in CHB patients before and after taking anti-HBV therapy. (c) Change in sperm motility and normal sperm morphologies during anti-HBV therapy. HBV: hepatitis B virus; CHB: chronic hepatitis B.

Thirty participants who met the anti-HBV criteria and were willing to accept anti-HBV nucleoside analog treatment were recruited to participate in the study. The mean age of patients was 32.2 years. Initial semen analysis was performed and 30 samples were all normal in terms of WHO 2010 criteria. Among the patients, 27 took ETV and 3 took LDT. The second semen analysis was conducted 6 months after drug administration. The tests showed that the semen volume (Figure 4(a)), semen concentration of sperm (Figure 4(b)), sperm motility, and normal sperm morphology (Figure 4(c)) were not changed significantly.

### 3.4. Consideration of Drug Choice for Child-Bearing Age Male Patients

**Figure 5:**
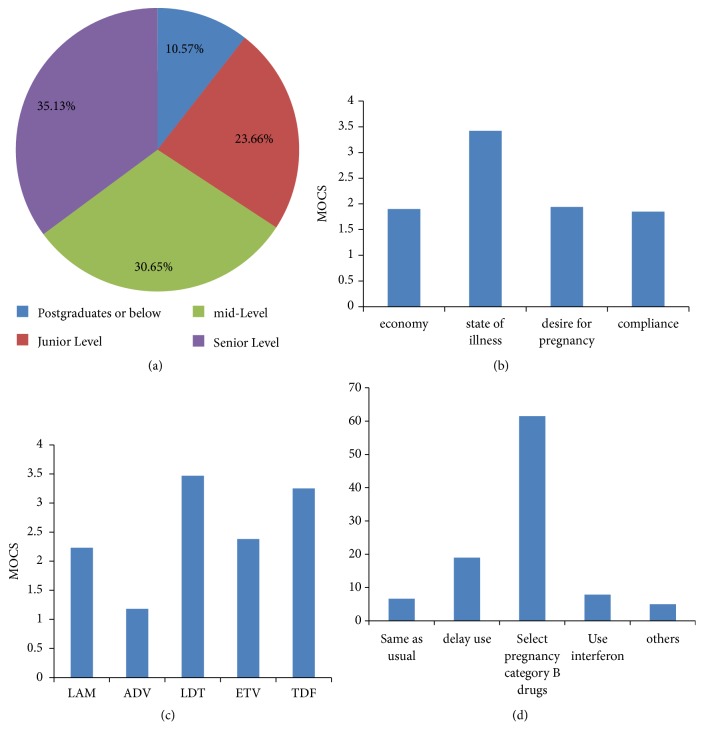
Consideration analysis of drug choice for child-bearing age male patients by doctors. (a) Professional composition of liver disease specialists who participated in questionnaire survey. (b) Considerations for setting a plan for male patients who met criteria for antiviral therapy. (c) Treatment selection for male patients who desired pregnancy in the near future. (d) Proportion of doctors with different recommendations for male patients who met criteria for antiviral therapy and desire future pregnancy. MOCS: mean options comprehensive score.

A total of 558 liver disease specialists nationwide participated in the questionnaire survey. Among them, 10.57% were postgraduate students, 23.66% were junior physicians, and 65.78% were mid-level physicians or above (Figure 5(a)). There were 51.25% male participants, and 48.57%, 29.93%, and 21.51% of the physicians consulted with ≥60%, 20-60%, and <20% HBV patients, respectively.

From the questionnaire survey of 558 liver disease specialists, the answer to “according to priority, what are the considerations when setting a plan for male patients who met criteria for antiviral therapy” question showed that 71.86% of the physicians considered state of illness as the basis for clinical administration, whereas 11.29% put “desire for pregnancy in the near future” as priority number two (Figure 5(b)). The answer to “for male patients who had desire for pregnancy in the near future, what are the priorities for treatment selection?” question showed that the first choice of drug was tenofovir disoproxil fumarate (37.28%), and the second was telbivudine (32.80%) (Figure 5(c)). The answer to “do you think antiviral nucleoside analogs for HBV therapy have an effect on male patient's fertility?” question indicated that 56.09% of the physicians were uncertain, 22.04% thought it had an effect, while 21.86% thought it had no effect. The answer to “what are the recommendations for male patients who met the criteria for antiviral therapy and have desire for pregnancy in the near future” question showed that 61.47% of physicians chose pregnancy category B antiviral nucleoside analogs for HBV treatment, whereas 19% chose to observe the patients' condition closely and delay antiviral therapy until childbirth (Figure 5(d)).

## 4. Discussion

By conducting a nationwide questionnaire survey of liver disease specialists and male HBV-positive patients in the Guangdong Province, we evaluated the current state of antiviral nucleoside analogs treatment on child-bearing age male HBV patients and physicians' considerations of antiviral therapy selection. We found that the main considerations for not administering antiviral therapy to CHB patients who met the criteria for antiviral therapy were drug effects on fertility, financial condition, compliance, and personal reasons. The effects of nucleoside analogs on pregnancy also influenced the treatment selection and timing of administration by physicians. More than half of the specialists were uncertain about the effects of nucleoside analogs on male fertility. The first choice of drug for patients with a desire for pregnancy was category B antiviral nucleoside analogs for HBV (LDT and TDF), while 19% of specialists recommended that patients delay antiviral treatment. Obviously, the desire for pregnancy influenced patients and physicians' awareness of the disease and its treatment.

The pharmacological mechanism of nucleoside analogs is based on the difference between the host cell and viral nucleic acid synthesis processes. Nucleoside analogs can selectively inhibit viral replication. Anti-HBV medicines are *β*-L-nucleosides with 3′-hydroxy, which can specifically inhibit hepadnaviridae without affecting human DNA polymerase and mitochondrial function [8]. Therefore, nucleoside analogs have high selectivity and low toxicity [9]. Most previous studies of nucleoside analog toxicity on fertility and pregnancy were conducted on pregnant mice. LAM showed no teratogenicity or effects on male and female fertility [10]. In rats and rabbits administered ADV orally (at approximately 23 and 40 times the human therapeutic dose of 10 mg/day, respectively), there was no toxicity on the placenta or teratogenicity [11].* In vivo* rat and rabbit studies showed no changes in male or female fertility following the administration of LDT [12]. Male rats administered TDF at 10 times the human recommended dose 28 days before mating and female rats administered TDF at 10 times the human recommended dose 15 days before mating showed no sexual function abnormalities, infertility, or birth defects [13]. Reproductive toxicology studies, in which animals were administered entecavir for up to 4 weeks, showed no evidence of impaired fertility in male or female rats. Although seminiferous tubular degeneration was evident in repeat-dose toxicology studies in rodents and dogs at exposures ≥26 times those in humans, no testicular changes were evident in a 1-year study in monkeys [14]. Our study showed no difference in conception rate and birth defect incidence rate between male patients who received antiviral nucleoside analogs in the preconception period and those who did not.

Male to female ratio of HBV patients was 6:1 [15]. According to China national epidemiology survey from 1992 to 1995, there were 40 HBsAg positive fathers with a total of 40 children, and the HBsAg positive rate in children was 15%. The infection rate in children of HBsAg-positive fathers was distinctively higher than that of children of HBsAg-negative parents (7.14%), indicating that paternal infection phenomenon exists [16]. Therefore, men who meet the antiviral therapy criteria should continue the antiviral therapy for protection from liver disease progression and to protect family members from HBV infection. Our study showed that the birth defect incidence rate was not higher in male patients who received antiviral therapy during preconception period. Additionally, no offspring had HBV infection.

According to 2015 prevention guidelines, for patients who meet the criteria for antiviral therapy, the first choice of nucleos(t)ide analogs is drugs with high barrier to resistance and strong antiviral activity such as ETV and TDF [17]. In our study, patients without desire for pregnancy mostly received ETV (73.96%), whereas only 16.67% received ETV as antiviral therapy among patients with desire for pregnancy. Male patients who had a desire for pregnancy were mostly administered LDT (62.50%). Physicians mostly recommended LDT and TDF, to child-bearing age male patients who needed antiviral therapy. Since TDF was introduced in China in 2014 and is not covered by medical insurance, it is not widely used among Chinese patients. Our study showed that 5.80% of patients without desire for pregnancy and 4.17% of patients with a desire for pregnancy received TDF. Therefore, for patients with a desire for pregnancy, LDT is the first choice, and the desire for pregnancy influenced physicians' drug selection.

Since there is no evidence to show that therapeutic dose of nucleos(t)ide analogs can cause sperm abnormalities and human body has a natural selection against abnormal zygotes, there was no evidence of nucleoside analog-related reproductive toxicity in male patients. According to our research, >56% of physicians were uncertain about the effects of anti-HBV nucleoside analogs on male patient's fertility and, thus, cautiously chose the therapy option. Preconception planning was an important consideration for therapy selection (first choice of pregnancy B category drugs or no medication) and timing of administration. Thus, male patients did not receive the best treatment regimen and missed the timing of medication. Further clinical research is required to evaluate the practicability and long-term effects of this treatment regime on patients.

This study was conducted among male HBV patients in several hospitals and liver disease specialists in multiple regions of China. However, since we used a retrospective questionnaire survey, there was a possibility of patients' recall bias. In addition, in the survey of physicians, provincial and hospital selection were not randomized and might have a certain bias. Determination of patients' sexual function was solely based on patients' personal assessment and requires further studies for confirmation.

## 5. Conclusions

In conclusion, our research suggested that antiviral nucleoside analogs have no fertility toxicity in male HBV patients and no fetal safety concerns. We propose that further clarification of child-bearing age male patients' treatment regimen in the prevention guidelines is required, and physicians should administer the best treatment regimen with optimal timing of administration according to the guidelines.

## Data Availability

No data were used to support this study.
